# Understanding the interplay of carbon and nitrogen supply for ectoines production and metabolic overflow in high density cultures of *Chromohalobacter salexigens*

**DOI:** 10.1186/s12934-017-0643-7

**Published:** 2017-02-08

**Authors:** María J. Salar-García, Vicente Bernal, José M. Pastor, Manuel Salvador, Montserrat Argandoña, Joaquín J. Nieto, Carmen Vargas, Manuel Cánovas

**Affiliations:** 1 Departamento de Bioquímica y Biología Molecular B e Inmunología, Facultad de Química, Universidad de Murcia, Campus Regional de Excelencia Internacional “Campus Mare Nostrum”, 30100 Murcia, Spain; 2 Departamento de Microbiología y Parasitología, Facultad de Farmacia, Universidad de Sevilla, 41012 Seville, Spain; 3 Departamento de Ingeniería Química y Ambiental, Universidad Politécnica de Cartagena, Campus Regional de Excelencia Internacional “Campus Mare Nostrum”, Campus Muralla del MarCalle Doctor Fleming S/N, 30202 Cartagena, Spain; 4 Área de Biología, Dirección de Tecnología Química y Nuevas Energías, Centro de Tecnología de Repsol S.A., Ctra. de Extremadura A-5, Km. 18, 28375 Móstoles, Spain

**Keywords:** *C. salexigens*, Ectoines, Nitrogen assimilation, Carbon overflow, Halophilism, Fed-batch

## Abstract

**Background:**

The halophilic bacterium *Chromohalobacter salexigens* has been proposed as promising cell factory for the production of the compatible solutes ectoine and hydroxyectoine. This bacterium has evolved metabolic adaptations to efficiently grow under high salt concentrations by accumulating ectoines as compatible solutes. However, metabolic overflow, which is a major drawback for the efficient conversion of biological feedstocks, occurs as a result of metabolic unbalances during growth and ectoines production. Optimal production of ectoines is conditioned by the interplay of carbon and nitrogen metabolisms. In this work, we set out to determine how nitrogen supply affects the production of ectoines.

**Results:**

*Chromohalobacter salexigens* was challenged to grow in media with unbalanced carbon/nitrogen ratio. In *C. salexigens*, overflow metabolism and ectoines production are a function of medium composition. At low ammonium conditions, the growth rate decreased importantly, up to 80%. Shifts in overflow metabolism were observed when changing the C/N ratio in the culture medium. ^13^C-NMR analysis of ectoines labelling revealed a high metabolic rigidity, with almost constant flux ratios in all conditions assayed. Unbalanced C/N ratio led to pyruvate accumulation, especially upon N-limitation. Analysis of an *ect* ^−^ mutant demonstrated the link between metabolic overflow and ectoine biosynthesis. Under non ectoine synthesizing conditions, glucose uptake and metabolic overflow decreased importantly. Finally, in fed-batch cultures, biomass yield was affected by the feeding scheme chosen. High growth (up to 42.4 g L^−1^) and volumetric ectoine yields (up to 4.21 g L^−1^) were obtained by minimizing metabolite overflow and nutrient accumulation in high density cultures in a low nitrogen fed-batch culture. Moreover, the yield coefficient calculated for the transformation of glucose into biomass was 30% higher in fed-batch than in the batch culture, demonstrating that the metabolic efficiency of *C. salexigens* can be improved by careful design of culture feeding schemes.

**Conclusions:**

Metabolic shifts observed at low ammonium concentrations were explained by a shift in the energy required for nitrogen assimilation. Carbon-limited fed-batch cultures with reduced ammonium supply were the best conditions for cultivation of *C. salexigens*, supporting high density growth and maintaining high ectoines production.

**Electronic supplementary material:**

The online version of this article (doi:10.1186/s12934-017-0643-7) contains supplementary material, which is available to authorized users.

## Background

Compatible solutes are compounds that are accumulated in the cytoplasm of microorganisms to provide osmotic balance when exposed to osmotic stress [1, 2]. They are very soluble, low molecular weight, uncharged or zwitterionic organic compounds. Among compatible solutes, ectoine and its hydroxylated derivative hydroxyectoine are used as protectors of cells and tissues in pharmaceutical and cosmetic preparations (e.g. for skin protection) and stabilizers of enzymes and antibodies [3]. Novel applications are underway, especially in biomedicine and biotechnology.

The chemical synthesis of ectoines is unaffordable at the industrial level due to the high cost of precursors (such as diaminobutyrate) and the chirality of the molecule, which makes microbial production the most feasible alternative [4]. Ectoines biosynthesis shares the first steps with the aspartate family amino acid pathway [3, 5]. This pathway poses a challenge to cell homeostasis, since it is highly demanding in terms of carbon skeletons, metabolic energy and reducing equivalents, and evolution has driven the metabolism of natural producers towards optimality [6, 7].

Industrially acceptable microbial processes must have high yields and productivities. The development of efficient and economic biological processes with extremophiles is challenging due to both the technical difficulties associated with bioreactor operation [8] and the incomplete understanding of the physiology of the producing strains [3]. This may lead to inefficient transformation of feedstocks, due to secondary products and low biomass yields due to growth inhibition [8, 9].

At the industrial level, ectoines are produced by the “bacterial milking” process, involving cycles of fed-batch fermentation of *Halomonas elongata* at 10–15% (w/v) NaCl to allow ectoine accumulation, followed by osmotic downshock at 2–3% (w/v) NaCl to release the osmolytes from the cells [4, 10, 11]. *H. elongata* has been engineered to increase ectoine productivity by deleting the ectoine uptake system and the degradation pathway [4]. *Chromohalobacter salexigens* has been engineered to improve hydroxyectoine production at low temperature and salinity [12]. Alternative processes for the production of osmolytes are based on fed-batch fermentation of *Brevibacterium epidermis* [13] or *Halomonas boliviensis* [14], continuous synthesis and excretion of osmolytes by recombinant mesophilic bacteria such as *Escherichia coli* [15–17] and *Corynebacterium glutamicum* [18] or halophilic bacteria such as *Halomonas salina* [19, 20]. On the other hand, *Marinococcus* M52 grown in a fed-batch microfiltration system provided elevated concentrations of hydroxyectoine [21].

*Chromohalobacter salexigens* (formerly, *H. elongata*) is a model halophilic γ-proteobacterium which accumulates ectoines as major compatible solutes [22]. It has been proposed as an alternative ectoine(s) producer due to the extensive knowledge on its physiology and genetics, the availability of molecular tools for genetic manipulation and a sequenced genome [23]. The synthesis of ectoines is regulated to cope with high environmental salt concentrations and other abiotic stress conditions such as high temperature [24–26]. We have recently described the adaptations undergone by its carbon metabolism to cope with high osmolarity. In this bacterium, glucose metabolism efficiency is a function of salt concentration. Ectoines biosynthesis at high salinity acts as a carbon sink and cell growth is slowed down due to the burden imposed. Moreover, overflow metabolism is minimized and biomass yield increases [6, 25]. At high salinity the cells balance their nitrogen content and ectoines are accumulated at the expense of proteins. Recent contributions to the field have taken advantage of metabolic modeling. A detailed reconstruction of the metabolism of ectoines in *H. elongata* was been recently published [7]. The genome-scale reconstruction of the metabolism of *C. salexigens* [27] is not complete, since relevant pathways (such as ectoines catabolism) were not included and further discrepancies with experimental data have been identified (F. Piubeli, M. Argandoña, M. Salvador, J.J. Nieto and C. Vargas, personal communication).

Ectoines are a challenging target for metabolic engineers, since optimal production is conditioned by carbon and nitrogen metabolisms, and the interplay of nitrogen metabolism and ectoines production is unknown. In this work, we set out to determine its relevance for the production of ectoines. *C. salexigens* was challenged to grow in media with unbalanced carbon/nitrogen ratio. Shifts in growth, ectoines yield and metabolism were analyzed and used to design an efficient feeding scheme for fed-batch cultures. This study contributes to disclosing the principles below the efficiency of *C. salexigens* as an ectoines cell factory.

## Methods

### Bacterial strains and culture conditions

*Chromohalobacter salexigens* CHR61, a rifampicin resistant spontaneous mutant of *C. salexigens* DSM 3043^T^ strain, and the ectoine synthesis deficient strain *C. salexigens* CHR62 [28] (referred in the text as *ect* ^−^) were used throughout this study.

Precultures were grown in SW-2 medium containing 2% (wt/vol) of total salts: 15.6 g L^−1^ NaCl, 4.07 g L^−1^ MgSO_4_·7H_2_O, 2.6 g L^−1^ MgCl_2_·6H_2_O, 0.4 g L^−1^ KCl, 67 mg L^−1^ CaCl_2_·2H_2_O, 47 mg L^−1^ NaBr and 13 mg L^−1^ NaHCO_3_ and 5 g L^−1^ of yeast extract [29]. Cultures were started from frozen 20% glycerol stock cultures.

Glycerol stocks, solid culture media and precultures were supplemented with filter-sterilized rifampicine (Rf) to a final concentration of 25 μg mL^−1^.

For ectoines production and physiological studies, strains were cultured in M63 minimal medium (see below). Cultures were inoculated with late exponential phase SW-2 precultures, to an initial optical density (OD_600nm_) of 0.025 measured at 600 nm (Amersham Biosciences Novaspec Plus Visible Spectrophotometer, Uppsala, Sweden).

### Physiological studies

For physiological studies, cultures were grown in glucose M63 minimal medium (pH 7.2). Standard composition of medium was as follows: 20 mM, glucose, 16.3 g L^−1^ KH_2_PO_4_, 4.2 g L^−1^ KOH, 2 g L^−1^ (NH_4_)_2_SO_4_, 39.5 mg L^−1^ MgSO_4_·7H_2_O, 0.5 mg L^−1^ FeSO_4_·7H_2_O and 0.75–2.5 M NaCl [30]. *C. salexigens* CHR61 was grown with 0.75–2.5 M NaCl, while the ectoine mutant strain CHR62 was studied at 0.75 M NaCl since it is not able to grow in higher salinities [28]. Aerobic 100 mL batch cultures were grown in 0.5 L flasks incubated at 37 °C on a rotary shaker operated at 210 rpm.

The standard M63 medium was adapted by varying the concentrations of glucose and ammonium sulfate as described in the text, in order to analyze the effect of its composition on growth and ectoines production. Final glucose and ammonium concentration were varied to: (i) glucose concentration ranging from 10 to 100 mM with fixed 30 mM ammonium concentration and (ii) ammonium concentration ranging from 5 to 200 mM with a fixed 20 mM glucose concentration. Additionally, ammonium was substituted with 20 mM ectoine, alanine or glutamate to study the effect of organic nitrogen sources.

### Bioreactor cultures: batch and fed-batch cultivation

High cell density cultures were performed in a Biostat B fermenter (Braun, Melsungen, Germany) with a 2 L vessel. Oxygen and pH were monitored with electrodes (Mettler-Toledo, Greifensee, Switzerland). Dissolved oxygen was maintained over 30% saturation by controlling air flow and stirring between 1 and 4 vvm and 40–1200 rpm, respectively. The pH was kept at 7.2 by automated addition of HCl/KOH.

For fed-batch cultivation, cells were initially cultured in the Biostat B system in batch for 16–20 h, to mid-exponential phase (initial volume: 1 L). Then, cells were fed following an exponential regime with glucose as the limiting nutrient. The feeding rate was controlled in order to limit the growth rate to a set value [31]. For that aim, two concentrated medium feedings were designed with NaCl 2.5 M and 10- or 50-fold concentrated medium nutrients. The culture scheme was divided into two feeding phases consisting of 500 mL of the 10- and 50-fold medium, respectively, sequentially fed to the vessel. Final culture volume was 2 L.

### Analytical procedures

#### Cell growth

To monitor culture growth, cells were resuspended in a NaCl solution (0.75–2.5 M). Absorbance was measured at 600 nm (Amersham Biosciences Novaspec Plus Visible Spectrophotometer, Uppsala, Sweden). OD_600nm_ values and dry cell weight were correlated for the strain used. For that aim, samples were withdrawn at different times from cultures in M63 medium with 0.75 or 2.5 M NaCl. Cells were separated by filtration through 0.45 µm filters (Millipore Corp. Billerica, MA) and washed with a NaCl solution at the same concentration as in the growth medium. Filters were heat-dried to constant weight with Sartorius MA150 Moisture Analyzer (Sartorius AG, Göttingen, Germany). Cellular dry weight was correlated with OD_600nm_ values: X (g L^−1^) = 0.5572·OD (NaCl, 0.75 M); X (g L^−1^) = 0.5322·OD (NaCl 2.5 M).

#### Analysis of extracellular metabolites by ion exchange HPLC-RI/UV

Extracellular metabolites (glucose, gluconate, pyruvate, acetate, lactate) were analyzed by HPLC (Shimadzu Scientific Instruments, Columbia, MD), equipped with differential refractive index and diode array detectors (Shimadzu Scientific Instruments, Columbia, MD), using a cation-exchange column (HPX-87H, BioRad Labs, Hercules, CA). The mobile phase was 5 mM H_2_SO_4_ at 0.5 mL min^−1^ flow rate and 45 °C.

#### Extraction and analysis of intracellular metabolites by reverse phase HPLC–UV

Compatible solutes were extracted from cell pellets by a variation of the method of Blight and Dyer [32]. Briefly, cells were separated by centrifugation and stored at −20 °C until extracted. Cell pellets were dried in a Heto Vac VR-1 speedvac (Heto Lab Equipment, Allerod, Denmark), resuspended in 250 µL of methanol/chloroform/water mixture (10/5/4, v/v/v) and incubated for 15 min at 37 °C. To precipitate proteins and extract ectoines, 65 µL of chloroform and 65 µL of water were added. Liquid phases were separated by centrifugation and aqueous phase was separated, vacuum dried and reconstituted in H_2_O. Ectoine and hydroxyectoine were analyzed by HPLC as described elsewhere [6].

#### Spectrophotometric determination of glucose and ammonia

Glucose was determined by a glucose (HK) assay (Sigma Aldrich, Saint Louis, MO). Ammonium was analyzed by an assay based on glutamate dehydrogenase (R-Biopharm, Darmstadt, Germany). Measurements were performed in a 96-well microplate reader Synergy HT (Bio-Tek, Winooski, VT).

### Isotopic labeling studies using ^1^H-NMR and ^13^C-NMR

For the labeling experiments, cells were batch grown in M63 medium with 20 mM [2-^13^C]glucose (CortecNet, Voisins-Le Bretonneux, France). Cells were harvested by centrifugation (16,000×*g*, 15 min, 4 °C) in the mid to late exponential phase (OD_600nm_ 1.5–2). Compatible solutes (ectoines and glutamate) were extracted as previously explained. The aqueous extract was lyophilized and reconstituted in deuterated methanol. ^1^H-NMR and ^13^C-NMR spectra were recorded at 25 °C using Brucker AV400 and Brucker AV500 spectrometers at 400 and 500 MHz, respectively, and a relaxation time of 1 s in the case of ^1^H-NMR and 60 s for ^13^C-NMR. Peak areas were integrated for quantification. Flux ratios were estimated as previously described [6].

## Results

The M63 minimal medium used for physiological studies is optimized for *C. salexigens* balanced growth. In order to determine how this microorganism responds to perturbations in the carbon and nitrogen sources and identify dose–effect correlations, growth experiments were carried out by changing one individual medium component at a time while maintaining the other medium components constant. The effects of unbalanced carbon/nitrogen feeding on growth and ectoines production were assessed.

### Effect of glucose on growth and production of ectoines

**Fig. 1:**
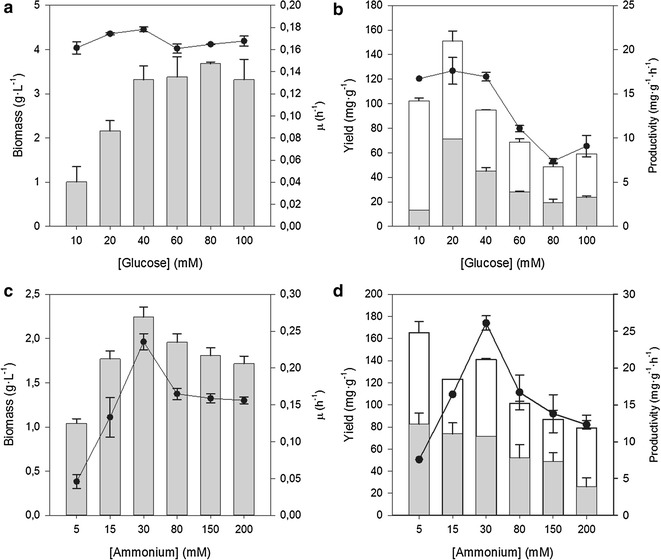
Effect of glucose and ammonium concentration on *C. salexigens* growth. The effect of glucose was analyzed in M63 medium with 30 mM ammonium and 10–100 mM glucose. **a** Growth rate (*dots*) and maximum biomass (*bars*), **b** specific ectoine (*grey bars*) and hydroxyectoine (*white bars*) yields; volumetric productivity of total ectoines (*black dots*). The effect of ammonium was analyzed in M63 medium with 20 mM glucose and 5–200 mM ammonium. **c** Growth rate (*dots*) and maximum biomass (*bars*), **d** specific ectoine (*grey bars*) and hydroxyectoine (*white bars*) yields; volumetric productivity of total ectoines (*black dots*). All experiments were performed at 2.5 M NaCl. *Error bars* denote the experimental deviation of at least three biological replicates. See the text for details

Batch growth experiments were performed at a fixed concentration of ammonium (30 mM), and a variable concentration of glucose (10–100 mM). At the concentrations tested, glucose did not affect the growth rate, while biomass yield increased with glucose concentration up to 40 mM, since the nitrogen source became limiting (Fig. 1a). At high glucose concentrations, the ectoine specific yields and productivities decreased by 50% (Fig. 1b), and the yield coefficient of biomass synthesis from glucose was also lower.

### Effect of ammonium on growth and production of ectoines

Batch growth experiments were performed at 5–200 mM ammonium and a fixed concentration of glucose (20 mM). At low ammonium concentrations, both the biomass yield and the growth rate increased proportionally with ammonium supply. Low biomass yields are caused by the limited synthesis of cellular components and ectoines upon nitrogen limitation. Additionally, the effect on the growth rate suggests that ammonium assimilation may be affected by its actual concentration in the growth medium. A slight decrease of growth rate was observed at high ammonium concentrations (Fig. 1c). The rate of cell decay in the stationary phase increased dramatically at high ammonium concentration (data not shown), which suggests that it might exert toxic effects, especially upon carbon source exhaustion. Ectoine content of cells decreased at high ammonium concentrations, while volumetric titer reached a maximum at 30 mM ammonium (Fig. 1d).

### Effects of ammonium availability on the metabolism of *C. salexigens*: carbon overflow and metabolic flux ratios

Unbalanced supply of glucose and ammonium might cause (i) metabolic shifts leading to overflow of by-products [6], or (ii) reduced availability of ammonium for ectoines and biomass synthesis. Inefficient growth correlated with the accumulation in the culture supernatant of gluconate, pyruvate and, to a lesser extent, acetate. The metabolite profiles were affected by the limiting nutrient. Gluconate and pyruvate accumulation was especially high in N-limited cultures, and continued after cell growth had been arrested. Metabolite overflow observed in C-limited cultures was much lower (Additional file 1: Table S1). At a constant initial glucose concentration, pyruvate production rate was greatly affected by ammonium availability, increasing proportionally to the C/N ratio in the medium.

**Fig. 2:**
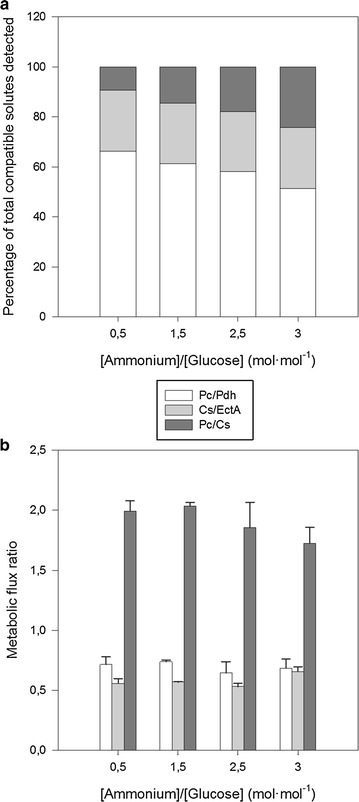
Effect of glucose or ammonium limitation on the metabolism of *C. salexigens.* Cells were grown at 2.5 M NaCl in M63 minimal medium with different ammonium to glucose ratios. [2-^13^C]-glucose was used as carbon source. The compatible solutes accumulated in the cells were extracted and the incorporation of the label at specific positions of the molecules was monitored using ^13^C-NMR and used to calculate metabolic flux ratios as described previously [6]. **a** Relative content of compatible solutes ectoine (*white bars*), hydroxyectoine (*grey bars*) and glutamate (*black bars*) in late exponential phase cells. **b** Selected metabolic flux ratios relevant for the functioning of the pyruvate node: pyruvate carboxylase (Pc) and pyruvate dehydrogenase (Pdh), citrate synthase (Cs) and ectoine synthase (EctA)

Metabolic ratios at relevant branching nodes related to the TCA cycle and the anaplerotic and ectoines biosynthesis pathways can be assessed from the incorporation of ^13^C from [2-^13^C]-glucose to specific positions on the backbone of ectoines [6]. Cells were fed with labelled glucose at different ammonium to glucose ratios and compatible solutes were analyzed by ^13^C-NMR (Additional file 2: Figure S1). At high ammonium to glucose ratio (i.e. in glucose limited cultures), glutamate accumulated to levels comparable to those of ectoines (Fig. 2a). Glutamate is a minor compatible solute in *C. salexigens*, and could result from glutamate dehydrogenase, a key branch-point enzyme between carbon and nitrogen metabolism [28]. Relevant metabolic flux ratios were estimated from the observed labelling patterns as described previously [6]. The ratio between pyruvate carboxylase and pyruvate dehydrogenase (Pc/Pdh), pyruvate carboxylase and citrate synthase (Pc/Cs) and citrate synthase and ectoine synthase (Cs/EctA) were estimated to be fairly constant in cultures with ammonium to glucose ratios from 0.5 to 3 (Fig. 2b). This reflects the high metabolic rigidity of *C. salexigens* when facing environmental perturbations.

### Linking metabolic overflow and ectoine metabolism: the *ect*^−^ mutant

Compatible solutes synthesis imposes a heavy metabolic burden which depends on salt concentration: at 2.5–3 M NaCl, ectoines make up to 16–24% of cell dry weight [6]. Given the connection of glucose and ammonia supply to ectoines synthesis and metabolic overflow, we wondered what would be the metabolic overflow if ectoines were not produced. For that aim, the metabolic profile of the wild type *C. salexigens* CHR61 strain was compared to that of the ectoine-deficient CHR62 mutant (CHR62 lacks the *ectABC* genes; thus, it will be denoted as *ect* ^−^ mutant herein).

**Fig. 3:**
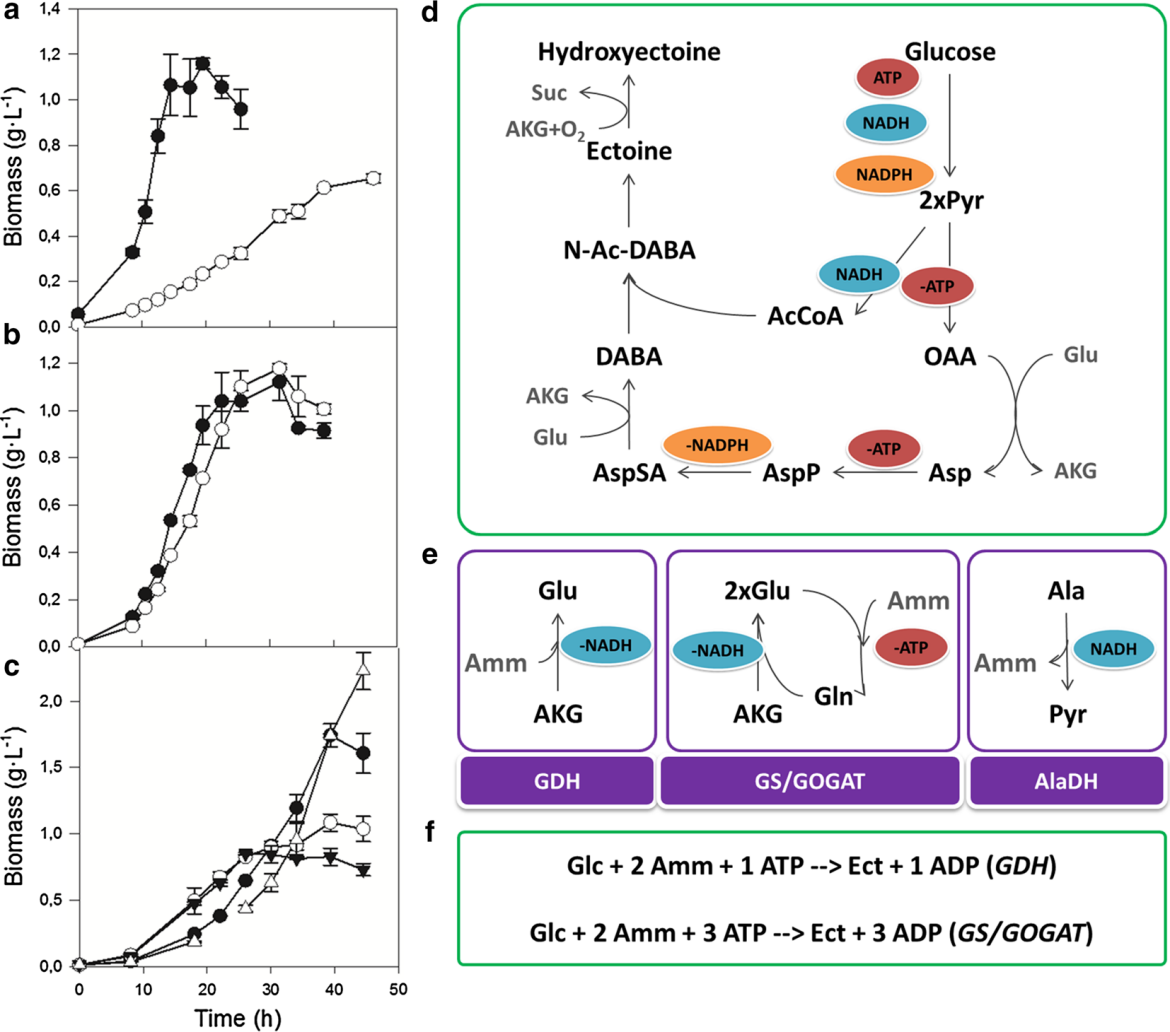
The link between nitrogen metabolism and ectoine synthesis. Effect of the *ect* ^−^ mutation on the growth of *C. salexigens*. **a** The strains CHR61 (wild type, *black symbols*) and CHR62 (*ect* ^−^ mutant, *white symbols*) were grown in M63 glucose minimal medium with 0.75 M NaCl. **b** The defective growth phenotype of the mutant CHR62 strain was recovered upon growth in M63 glucose minimal medium supplemented with 20 mM ectoine as the sole nitrogen source. **c** Effect of the nitrogen source selected for the growth of *C. salexigens.* The wild type CHR61 strain was grown in in M63 minimal medium with 2.5 M NaCl and supplemented with 30 mM ammonium (*black circles*), 20 mM alanine (*white triangles*), 20 mM glutamate (*black triangles*) or 20 mM ectoine (*white circles*) as the sole nitrogen source. **d** Link between central metabolism and the ectoines biosynthesis pathway in *C. salexigens* [6]. Cofactors produced and/or consumed in each pathway are indicated. Glucose is transformed into pyruvate by the Entner–Doudoroff pathway and ectoines are synthesized from oxaloacetate and acetyl-CoA. **e** Ammonium assimilation pathways in *C. salexigens*: glutamate dehydrogenase (GDH) and glutamine synthetase/glutamate synthase (GS/GOGAT). Alanine is catabolized by oxidative deamination catalyzed by alanine dehydrogenase (AlaDH)*. **f** Overall stoichiometry of ectoine biosynthesis in *C. salexigens* as a function of the ammonium assimilation pathway used. Ectoine biosynthesis from glucose leads to net consumption of 1 mol of ATP if ammonia is assimilated through GDH and 3 mol of ATP if it is assimilated through the GS/GOGAT pathway**. * Genes encoding glutamate dehydrogenase (*Csal1340*) and alanine dehydrogenase (*Csal2966*) have been annotated in the genome of *C. salexigens* [23]. Five genes encoding putative glutamine synthetases are annotated: *Csal0777, Csal1181, Csal1192, Csal0243, Csal0679*. Glutamate synthase is a heterodimeric protein composed of two different subunits encoded by *gltB* (*Csal0615*) and *gltD* (*Csal0616*) genes. ** Hydroxyectoine biosynthesis from glucose needs additionally 1 mol NADH and 1 mol GTP due to the transformation of α-ketoglutarate into succinate by ectoine hydroxylase

Growth of the *ect* ^−^ mutant is severely impaired and it only grows at moderate salt concentrations (e.g. 0.75 M NaCl) and at a much lower rate (Fig. 3a). When grown with 20 mM ectoine as the sole nitrogen source, both strains showed the same growth profile (Fig. 3b). This demonstrates that: (i) defective growth of the *ect* ^−^ mutant is due to its inability to synthesize ectoines and (ii) *C. salexigens* can use ectoines as the sole nitrogen source, uptake and assimilation being preferred over de novo synthesis.

Metabolic overflow was also assessed in the *ect* ^−^ mutant. When growing on ammonium as nitrogen source, the *ect* ^−^ mutant showed an inefficient metabolism, with a high glucose consumption coefficient (Y_Glc/X_) and a heavy accumulation of gluconate in the culture broth (Additional file 3: Table S2). Pyruvate and acetate overflow was similar for both strains, representing approximately 20% of the glucose consumed. Since the *ect* ^−^ mutant synthesized no ectoines, lower metabolic burden led to a 30% decrease in the glucose consumption rate. Despite this, gluconate accumulation rate was 9- to 12-fold higher, which suggests that in this mutant strain oxidation of glucose is not regulated to meet growth needs but, rather, responds to osmotic-stress [28] as demonstrated in mesophilic bacteria which do not produce compatible solutes [33, 34].

In the presence of 20 mM ectoine, both strains exhibited similar growth profiles. The specific rates of glucose consumption and pyruvate and gluconate production of wild type *C. salexigens* were lower than in the control medium (Additional file 3: Table S2). Thus, glucose consumption and production of gluconate and overflow metabolites are linked to the metabolic burden imposed by ectoine production. Acetate production increased, which might be result from ectoines degradation [7].

### Linking metabolic overflow and nitrogen metabolism: the effect of the nitrogen source on growth

To further confirm the link between nitrogen assimilation pathways and metabolic burden, the wild type *C. salexigens* strain was grown in minimal medium with 2.5 M NaCl with four nitrogen sources: ammonia, ectoine, alanine and glutamate. The amino acids alanine and glutamate were selected since both are transformed to compounds of central metabolism closely related to ectoines biosynthesis and serve as nitrogen sources.

Similar growth profiles were observed in the presence of ectoine or glutamate as nitrogen sources, with shorter *lag* phase than in control cultures with ammonium as the sole nitrogen source. These results suggest that ammonia assimilation is a substantial contributor to the metabolic burden imposed by ectoine biosynthesis. A prolonged lag was observed in the growth on alanine (Fig. 3c), probably due to the accumulation of pyruvate and ammonia, formed during alanine degradation [35, 36].

### Fed-batch cultivation of *C. salexigens*

In an attempt to find conditions to sustain high density cultivation and high ectoine titers, we decided to grow *C. salexigens* in glucose limited fed-batch cultures. The nutrient feeding profile was designed using glucose as the growth-limiting nutrient. After an initial batch phase, the culture was fed with a concentrated nutrient solution, fixing the growth rate through the feeding rate [31]. Growth, production of ectoines and metabolites, and nutrient consumption were profiled along cultures.

When growth rate was adjusted to a relatively high level (0.1 h^−1^), control of growth was poor, and glucose and ammonium accumulated, resulting in early growth arrest at a low cell density (12.6 g L^−1^) due to nutrient inhibition. Much higher growth (28.1 g L^−1^) was reached at a lower exponential feeding rate (0.05 h^−1^) (Table 1). In this case, glucose was the growth-limiting nutrient, and it was completely exhausted from the culture medium. However, ammonium feeding was not balanced and accumulated to high levels, finally leading to growth inhibition. In order to minimize this effect, ammonium concentration in the initial growth medium and in the feeding solutions was lowered. This strategy led to increased biomass yield. Best results were obtained when the initial concentration of ammonium was 15 mM (Table 1).

**Table 1:** Comparison of batch and fed-batch cultures of *C. salexigens* DSM 3043

	Batch	Fed batch A	Fed batch B	Fed batch C
Set μ (h^−1^)	N/A	0.1	0.05	0.05
Initial [glucose] (mM)	20	20	20	20
Initial [ammonium] (mM)	30	30	30	15
Maximum biomass (g L^−1^)	2.12 ± 0. 2	12.6 ± 1. 6	28.1 ± 3.0	42.4 ± 2.0
Ectoine production (g L^−1^)	0.16 ± 0. 02	0.27 ± 0. 02	1.62 ± 0. 2	1.96 ± 0. 6
Hydroxyectoine production (g L^−1^)	0.18 ± 0. 02	0.18 ± 0. 03	1.51 ± 0. 3	2.25 ± 0. 7
Overall (g_CDW_ g_glucose_^−1^)	0.59	0.39	0.50	0.76
Time (h)^a^	36	52	124	102
Stirring (rpm)	41–554	41–860	41–1138	41–1149
pO_2_ setting (%O_2_)	30	30	30	30
Aeration (L min^−1^)	1–6	1–4	1–2	1–3.5

Experiments were performed in duplicate (batch and fed-batch A) or triplicate (fed-batch B and C). Data are average of experimental replicates

^a^Time taken to reach maximum biomass

**Fig. 4:**
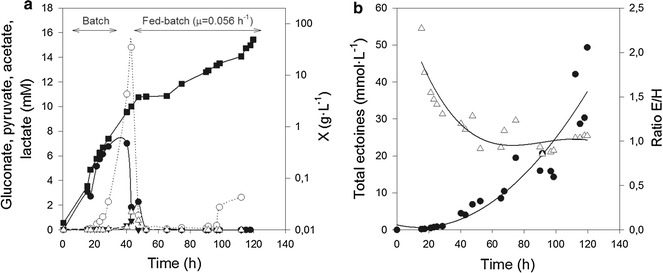
High density cultivation of *C. salexigens* in fed-batch system. **a** Culture profile of a fed-batch culture of *C. salexigens* exponentially fed at a fixed rate of 0.05 h^−1^. The initial batch and the fed-batch phases are indicated in the figure. The evolution of biomass (*black squares*) and the major by-products, gluconate (*black circles*), pyruvate (*white circles*), acetate (*black triangles*) and lactate (*white triangles*) is shown. **b** Time profile of volumetric titer of total ectoines (*black circles*), and ectoine to hydroxyectoine ratio (*white triangles*) in the same fed-batch culture. Fermentations were run in a Biostat B system, as described under the “Methods” section under conditions denoted as Fed-Batch C (see Table 1 for details on conditions)

Exponential feeding with a “low nitrogen” nutrient solution in which glucose was the growth limiting nutrient, also allowed limiting metabolic overflow. Accumulation of gluconate and pyruvate was observed during the initial phases of cultures (up to 7 and 15 mM, respectively). Much lower amounts of acetate and lactate were observed. From 50 h of culture onwards, none of these metabolites was detected in the culture supernatants (Fig. 4), indicating a truly C-limited regime with cells efficiently scavenging by-products to maximize metabolic yields. In fact, two culture phases can be distinguished in the growth profile. Specific growth rate in the first phase was typical of batch regime (0.16 ± 0.03 h^−1^). As the fed-batch phase started and glucose became limiting, growth rate was controlled at 0.056 ± 0.003 h^−1^, demonstrating a true fed-batch control of growth.

Moreover, the yield coefficient calculated for the transformation of glucose into biomass was higher than in the batch culture. This demonstrates that overflow metabolism observed in batch cultures can be minimized in C-limited fed-batch, which is an indication that the metabolic efficiency of this microorganism can be improved by careful design of culture feeding schemes.

## Discussion

In this work we have shown the close relationship between nitrogen assimilation and ectoine production in *C. salexigens*. Engineering microbes for the production of nitrogenated compounds involve additional challenges, since both carbon and nitrogen metabolisms have to be rewired. Several cases of success have been reported, most of them for the biosynthesis of amino acids [37, 38].

While the growth rate of *C. salexigens* is not responsive to the concentration of glucose, it is greatly affected by ammonium. Both high and low ammonium concentrations affect growth and, interestingly, also ectoine production. In order to understand this behavior, central metabolism and the ectoine production pathway have to be jointly considered. For bacteria grown with ammonium as the nitrogen source, the building blocks for the biosynthesis of proteins, nucleic acids and all other nitrogen-containing cellular components derive from glutamate and glutamine [36]. Two major systems have been described in bacteria for the incorporation of ammonium to the backbone of α-ketoglutarate (Fig. 3e). These pathways differ in substrate affinity and overall energy balance. The glutamine synthetase glutamate synthase (GS/GOGAT) system is the main pathway at low (<1 mM) ammonium concentrations, while glutamate dehydrogenase (GDH) is the main system at high concentrations. The GS/GOGAT system consumes one ATP equivalent per glutamate molecule synthesized [36].

The synthesis of ectoine and hydroxyectoine consumes glutamate as the donor of amino groups for the synthesis of aspartate (through aspartate aminotransferase) and l-2,4-diaminobutyrate (through EctB) (Fig. 3d) [5]. In silico analysis of the metabolic network of *H. elongata* underlined that the use of either of these systems for ammonium uptake alters the overall stoichiometry of ectoine synthesis [7]. In *C. salexigens*, which metabolizes glucose through the poor ATP-yielding Entner–Doudoroff pathway, this effect is even more pronounced, and ectoine synthesis leads to net consumption of ATP both at high and low ammonium concentration conditions. This is especially dramatic at low ammonium concentrations, since the GS/GOGAT pathway spends more cellular energy for nitrogen assimilation, lowering growth efficiency and forcing cells to balance the extra energy required to produce ectoines (Fig. 3f). At higher ammonium concentrations, ammonium is assimilated through the energetically less expensive GDH pathway.

Metabolism of *C. salexigens* adapts dynamically to varying energy requirements. Metabolic overflow was particularly sensitive to variations in ammonium availability; pyruvate and acetate are overflow metabolites, serving as readout of inefficient metabolism. Gluconate cannot be considered an overflow metabolite since it is produced by fast oxidation of glucose, an adaptation to gain advantage in the competition for carbon sources with microorganisms which are unable to use gluconate as carbon source [39]. At a constant initial glucose concentration, pyruvate production rate increased proportionally to the C/N ratio in the medium, i.e. was higher as ammonium became more limiting. This suggests that at low ammonium conditions, glucose is incompletely oxidized to pyruvate to yield fast ATP to support the ATP-expensive ectoines biosynthesis pathway. Interestingly, this occurred without affecting central metabolism flux ratios reflecting the activity of the TCA cycle and the anaplerotic and ectoines biosynthesis pathways. A similar metabolic rigidity was previously observed in the response of *C. salexigens* to salt concentration [6] and it is further evidenced here in response to perturbations in the supply of nitrogen source. Apparently, environmental pressure has evolved a rigid metabolism to favor high ectoines biosynthetic fluxes at the expense of other adaptability strategies. Overflow metabolism may serve other roles. For instance, pyruvate overflow might feed other microbes in hypersaline environments [40].

In addition to their role as protecting agents, compatible solutes also are storage materials which can be used as carbon and nitrogen sources by microbes when environmental stresses decrease. This is also the case of ectoines, which can be used as carbon [3] and nitrogen sources (this work). Ectoines catabolism has been described in *C. salexigens* [41], *Sinorhizobium meliloti* and *H. elongata* [7] and *Ruegeria pomeroyi* [42]. In *C. salexigens* and *S. meliloti*, it occurs through at least two systems with high similarities. Jebbar and col. identified an ectoine-induced operon involved in the uptake and catabolism of ectoine in *S. meliloti* (*ehuABDC*-*eutABCD*) [43]. In *C. salexigens*, orthologues to some of these genes have been found, although their genomic organization is different [3]. Ectoines catabolism yields acetate as end product. In fact, acetate was detected in the supernatants of ectoine supplemented cultures of wild type and *ect* ^−^ *C. salexigens* strains. Moreover, acetate overflow in *C. salexigens* is higher at low salt concentration [6], which might suggest that ectoines degradation might be regulated, while ectoines synthesis would be semi-constitutive. This hypothesis would explain the constant flux ratios assessed at different salinities [6].

The amino acids alanine and glutamate were selected for the nitrogen source experiments since both are readily metabolized yielding intermediaries of central metabolism, and nitrogenated precursors of ectoines synthesis [44]. l-Glutamate is a nitrogen source for ectoines synthesis: transamination of l-glutamate yields l-aspartate (which is the precursor of ectoines synthesis) [3, 44–46] and l-glutamate is the nitrogen donor for the l-aspartate-β-semialdehyde to l-2,4-diaminobutyrate transformation (catalyzed by EctB) [3, 47]. In fact, glutamate is used as nitrogen source for the growth of many halophiles (see for instance [14]). In bacteria, l-alanine degradation occurs through oxidative deamination to pyruvate and ammonium [35]. In *C. salexigens*, Csal2966 gene is annotated as alanine dehydrogenase [44]. This reaction generates free ammonium to be used as nitrogen source [36], which assimilation is highly affected by its concentration in the medium (this work). This would explain why growth was delayed after M63 medium supplementation with alanine.

Although optimizing the production process was not the objective, the titers and productivities of ectoine reported herein are close to those previously reported with other halophiles [3, 14]. The best performance of *C. salexigens* was reported by Fallet et al. [48] using a continuous cultivation system consisting of a cascade of two continuously operated bioreactors. High cell density growth and ectoines production occurred in the first bioreactor, using cross-flow ultrafiltration for biomass retention. In the second bioreactor the cell broth was concentrated and subjected to an osmotic down-shock to force secretion of ectoines. With this system, they reported up to 61 g L^−1^ of biomass, 8.2 g L^−1^ of ectoines and a productivity of 2.1 g L^−1^ h^−1^.

Higher productivities of ectoines were assessed in cultures fed with low glucose and moderate ammonia levels. *C. salexigens* is adapted to thrive in nutrient poor environments, a phenomenon common to many other microorganisms, which usually exhibit high carbon overflow during growth on nutrient rich media. Low nutrient fluxes limit biomass produced, thus decreasing process productivity, especially with low nitrogen levels, which has an effect on the growth rate. Pyruvate and acetate overflow and gluconate accumulation might be avoided to ensure high productivities, and high biomass and specific ectoines yields should be reached to attain high levels of ectoines. In fed-batch cultures, growth rate was controlled using glucose as the limiting nutrient and ammonium content in the broth was adjusted in order to keep it within moderate values. The metabolism of *C. salexigens* was manipulated to avoid the activation of the ATP-expensive GS/GOGAT ammonium assimilation pathway and minimizing overflow metabolism. In fact, the overflow metabolism was only observed in the initial batch growth phase. Thus, further understanding the physiology of cell factories allows rationally developing high density fed-batch cultures and maximizing process productivities.

## Conclusions

In this work, the importance of balanced carbon and nitrogen fluxes for optimal production of ectoines is demonstrated. The metabolism of *C. salexigens* is optimized to maximize the survival of the microorganism in nutrient limited systems and is highly inefficient in nutrient-rich environments, growth yield being limited by carbon overflow. The overflow metabolism shifts observed in *C. salexigens* with varying C/N ratios did not correlate with changes in flux ratios demonstrating the metabolic rigidity of *C. salexigens*. Finally, high density *C. salexigens* cultivation was possible with a low nitrogen feeding.

## Additional files

**Additional file 1: Table S1.** Effect of carbon and nitrogen feeding ratios on metabolic overflow in *C. salexigens*. Cells were cultured using M63 medium supplemented with glucose and ammonium at the concentrations shown in the Table.

**Additional file 2: Figure S1.** (A) ^13^C-NMR spectra of ectoines produced by *C. salexigens* in the presence of different ammonium concentrations in the growth medium. Cells were grown in M63 minimal medium with 2.5 M NaCl. The concentration of ammonium was 30 mM (upper spectrum) and 50 mM (lower spectrum). See the “Methods” section for details. In the inserts, the signals from Glu, C5 and Ect, C1 (B) and Glu, C3 and Ect, C5 (C) are shown. The color schemes represent the expected labelling pattern of ectoine (Ect) and glutamate (Glu). These schemes represent all different possible routes from which ectoines backbones can be derived: (d) pyruvate dehydrogenase (Pdh) *plus* one TCA cycle turn, (e) pyruvate carboxylase (Pc), (f) pyruvate carboxylase (Pc) *plus* one TCA cycle turn. The color code indicates *per* cent labelling at each carbon position, according to the color scale shown in the left bar. When the ball is split in two, upper half corresponds to anaplerosis through Pc (or Ppc) from pyruvate (or PEP) labelled at C2 and the lower half corresponds to anaplerosis from unlabeled PEP.

**Additional file 3: Table S2.** Growth of *C. salexigens* CHR61 (wild type) and CHR62 (*ect* ^−^ mutant) in M63 minimal medium with 0.75 M NaCl.
