# Supplementary material for: Understanding the interplay of carbon and nitrogen supply for ectoines production and metabolic overflow in high density cultures of Chromohalobacter salexigens
Source: Microb Cell Fact. 2017 Feb 8;16:23. doi: 10.1186/s12934-017-0643-7 (PMC5299690; doi:10.1186/s12934-017-0643-7)
Supplement: Supplementary file 1 — Additional file 1: Table S1. Effect of carbon and nitrogen feeding ratios on metabolic overflow in C. salexigens. Cells were cultured using M63 medium supplemented with glucose and ammonium at the concentrations shown in the Table. [file 12934_2017_643_MOESM1_ESM.docx]

**Table S1. Effect of carbon and nitrogen feeding ratios on metabolic overflow in *C. salexigens*. Cells were cultured using M63 medium supplemented with glucose and ammonium at the concentrations shown in the Table.**

|  | **[Glc] (mM)** | **[Amm] (mM)** | **qGlcnt*** | **qPyr*** | **qAc*** |
| --- | --- | --- | --- | --- | --- |
| **Variable glucose** | 10 | 30 | 4.74 ± 0.03 | 1.49 ± 0.13 | N.D. |
|  | 20 | 30 | 2.44 ± 0.36 | 6.51 ± 0.18 | N.D. |
|  | 40 | 30 | 5.14 ± 1.23 | 4.97 ± 0.23 | N.D. |
|  | 60 | 30 | 10.72 ± 2.51 | 5.73 ± 2.30 | N.D. |
|  | 80 | 30 | 2.83 ± 0.50 | 7.13 ± 0.76 | N.D. |
|  | 100 | 30 | 5.37 ± 0.61 | 6.63 ± 0.25 | N.D. |
| **Variable ammonium** | 20 | 5 | 40.74 ± 5.08 | 43.33 ± 9.83 | N.D. |
|  | 20 | 15 | 1.61 ± 0.01 | 15.77 ± 1.42 | 2.13 ± 0.21 |
|  | 20 | 30 | 2.44 ± 0.36 | 6.51 ± 0.18 | N.D. |
|  | 20 | 80 | 5.66 ± 2.35 | 2.38 ± 0.23 | N.D. |
|  | 20 | 150 | 3.66 ± 1.44 | 1.39 ± 0.71 | N.D. |
|  | 20 | 200 | 8.26 ± 1.62 | 1.60 ± 0.18 | N.D. |

* Rates expressed as mmol·g_DCW_^-1^·h^-1^.

*N.D. Not detected.*
