# Supplementary figures and images for: Understanding the interplay of carbon and nitrogen supply for ectoines production and metabolic overflow in high density cultures of Chromohalobacter salexigens
Source: Microb Cell Fact. 2017 Feb 8;16:23. doi: 10.1186/s12934-017-0643-7 (PMC5299690; doi:10.1186/s12934-017-0643-7)

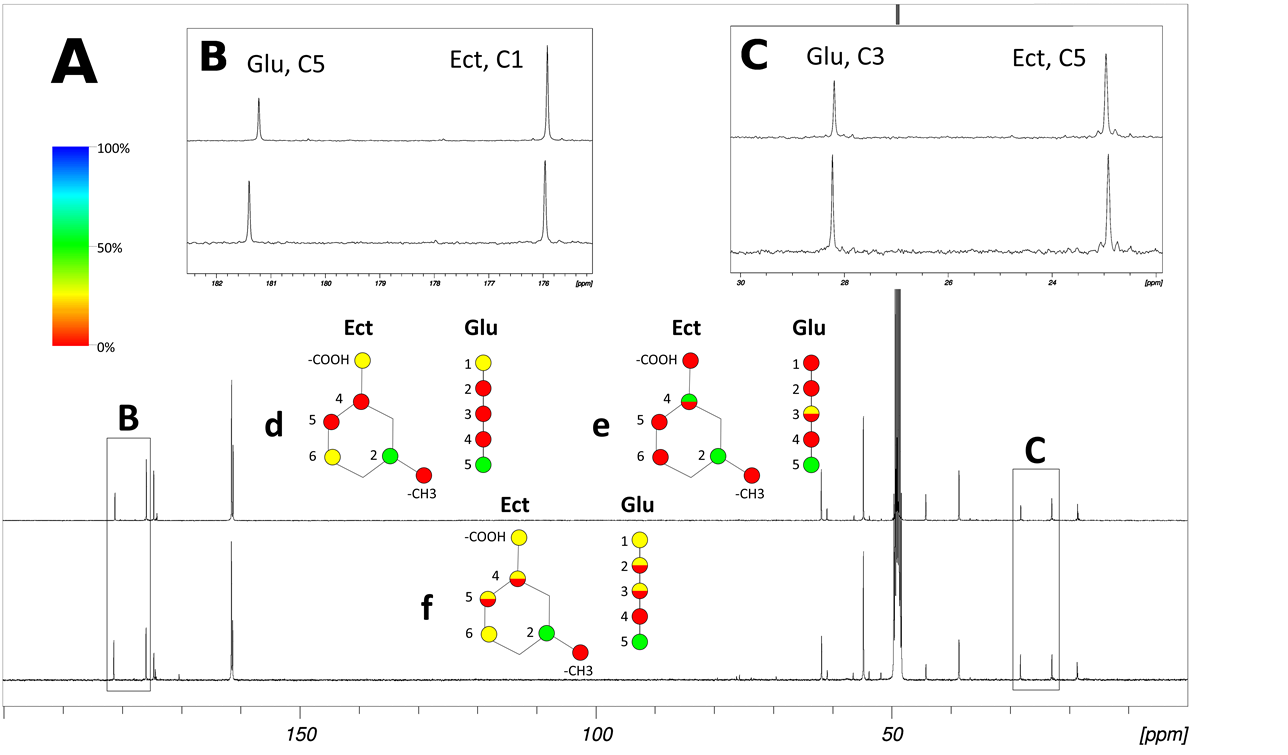

Supplement: Supplementary file 2 — Additional file 2: Figure S1. (A) 13C-NMR spectra of ectoines produced by C. salexigens in the presence of different ammonium concentrations in the growth medium. Cells were grown in M63 minimal medium with 2.5 M NaCl. The concentration of ammonium was 30 mM (upper spectrum) and 50 mM (lower spectrum). See the “Methods” section for details. In the inserts, the signals from Glu, C5 and Ect, C1 (B) and Glu, C3 and Ect, C5 (C) are shown. The color schemes represent the expected labelling pattern of ectoine (Ect) and glutamate (Glu). These schemes represent all different possible routes from which ectoines backbones can be derived: (d) pyruvate dehydrogenase (Pdh) plus one TCA cycle turn, (e) pyruvate carboxylase (Pc), (f) pyruvate carboxylase (Pc) plus one TCA cycle turn. The color code indicates per cent labelling at each carbon position, according to the color scale shown in the left bar. When the ball is split in two, upper half corresponds to anaplerosis through Pc (or Ppc) from pyruvate (or PEP) labelled at C2 and the lower half corresponds to anaplerosis from unlabeled PEP. [file 12934_2017_643_MOESM2_ESM.png]
