# Supplementary material for: Understanding the interplay of carbon and nitrogen supply for ectoines production and metabolic overflow in high density cultures of Chromohalobacter salexigens
Source: Microb Cell Fact. 2017 Feb 8;16:23. doi: 10.1186/s12934-017-0643-7 (PMC5299690; doi:10.1186/s12934-017-0643-7)
Supplement: Supplementary file 3 — Additional file 3: Table S2. Growth of C. salexigens CHR61 (wild type) and CHR62 (ect − mutant) in M63 minimal medium with 0.75 M NaCl. [file 12934_2017_643_MOESM3_ESM.docx]

**Table S2. Growth of *C. salexigens* CHR61 (wild type) and CHR62 (*ect*^-^ mutant) in M63 minimal medium with 0.75 M NaCl.**

| **Medium** | **Strain** | **µ (h^-1^)** | **qGlc^a^** | **Y_Glc/X_^b^** | **qGlcnt^a^** | **qPyr^a^** | **qAc^a^** |
| --- | --- | --- | --- | --- | --- | --- | --- |
| **M63 (0.75 M NaCl)** | Wild type | 0,20±0.01 | 3.52±0.40 | 17.53±2.00 | 0.25±0.12 | 1.06±0.08 | 0.31±0.04 |
|  | CHR62 | 0.10±0.01 | 2.59±0.14 | 25.92±1.40 | 2.42±0.11 | 0.72±0.07 | 0.32±0.02 |
| **M63 (0.75 M NaCl)**  **Suppl. 20 mM Ect** | Wild type | 0.18±0.01 | 2.55±0.05 | 14.19±0.26 | 0.01±0.01 | 0.12±0.01 | 0.68±0.03 |
|  | CHR62 | 0.19±0.01 | 2.84±0.23 | 14.97±1.21 | 0.12±0.06 | 1.01±0.21 | 0.93±0.06 |

**^a^** Rates expressed as mmol·g_DCW_^-1^·h^-1^

^b^ Yield coefficients expressed as mmol·g_DCW_^-1^.
